# Indonesian sign language system (SIBI) dataset: Sentences enhanced by diverse facial expressions for total communication

**DOI:** 10.1016/j.dib.2025.111642

**Published:** 2025-05-10

**Authors:** I Dewa Made Bayu Atmaja Darmawan, Linawati, Gede Sukadarmika, Ni Made Ary Esta Dewi Wirastuti, Reza Pulungan

**Affiliations:** a Doctoral Engineering Department, Udayana University, Denpasar, Indonesia; b Electrical Engineering Department, Udayana University, Badung, Indonesia; c Department of Computer Science and Electronics, Universitas Gadjah Mada, Yogyakarta, Indonesia

**Keywords:** Sign language, SIBI, Hand gestures, Facial expression, Total communication, Video data

## Abstract

Total communication in sign language is a communication approach that involves several components in conveying information, including hand movements, body movements, lip movements, and facial expressions. Currently, there is no publicly available Indonesian Sign Language (SIBI) dataset. Therefore, researchers must collect their own data. Utilizing diverse datasets can result in partiality when making inferences about the suggested approach. The SIBI dataset was built by involving 10 teachers and 10 hearing-impaired high school students. The SIBI dataset includes basic signs and sign sentences. Basic sign gestures include letters, numbers, and affixes, according to the SIBI online dictionary published by the Indonesian Government. Sentences are created by several teachers using common communication phrases during learning process. Sign language sentences are delivered using total communication, which combines lip movements, body movements, and facial expressions. The same sentence can be delivered with different facial expressions, giving different meanings. This article discusses how a sign sentence is expressed with different facial expressions. In SIBI, word morphology follows the rules of the Indonesian language. Therefore, this paper also explains the morphemic structure of the 40 sentences used as references. The use of the SIBI dataset can be adjusted to certain research parameters. The video dataset can be converted into a series of frames or images as needed for the research. Although audio is not available, the SIBI video data captures lip movements that can be used in lip reading research. The SIBI dataset can facilitate research in computer vision as well as other fields of study related to sign language.

Specifications Table

**Table:** 

Subject	Computer Vision and Pattern Recognition.
Specific subject area	*Sign Language Recognition*.
Type of data	Video (.mp4), Text (.csv).
Data collection	The video data was recorded in a controlled environment, using a green background with adjusted lighting. The signer was given guidance before recording. During recording, instructions and examples of movements were shown to ensure that every movement made by the signer was uniform. Video recording used a Logitech C920 with a resolution of 1920×1080 and a frame rate of 30 fps. The dataset was then built by cropping to 800×800 using Wondershare Filmora.
Data source location	• Institution: SLB Negeri 2 Denpasar. • City/Province: Denpasar/Bali. • Country: Indonesia. • Latitude and longitude: 8°42′ 10.48′′ S, 115°13′ 43.329′′ W.
Data accessibility	Repository name: Indonesian Sign Language System (SIBI) Dataset Data identification number: 10.17632/44pbrbsnkh.3 Direct URL to data: https://data.mendeley.com/datasets/44pbrbsnkhWe publish metadata (CSV) and a video of 1 subject (author) with a blurred face. To obtain the full version of the dataset, which consists of 20 subjects with visible faces, researchers must understand and agree to the Data Use Agreement (DUA). We include the DUA as supplementary material and attach it to the dataset link. The DUA is provided in two options, Indonesian and English. Student researchers must include an approval/recommendation sheet from their research supervisor. The data owner will send a link to the full version of the SIBI Dataset after receiving the signed DUA file.
Related research article	[1] I.D.M.B.A. Darmawan, Linawati, G. Sukadarmika, N.M.A.E.D. Wirastuti, R. Pulungan, Mulyanto, N.K.D. Hariyanti, Advancing Total Communication in SIBI: A Proposed Conceptual Framework for Sign Language Translation, 2023 International Conference on Smart-Green Technology in Electrical and Information Systems (ICSGTEIS), (2023) 23–28. 10.1109/ICSGTEIS60500.2023.10424020.

## 1 Value of the Data

• This dataset refers to the SIBI online dictionary^1^ released by the Ministry of Education and Culture of the Republic of Indonesia, using 20 signers consisting of 10 special school teachers and 10 hearing-impaired high school students.
• The dataset consists of basic gesture forms, including the alphabets, numbers, and affixes, as well as more complex forms, consisting of 40 sentences conveyed in 7 different facial expressions.
• The dataset can be helpful for various parties, including researchers, teachers, or those who want to learn sign language.
• The Indonesian Sign Language System (SIBI) differs from general sign language, which typically has distinct grammar from spoken language. SIBI, however, follows the Indonesian grammar.
• The sentence dataset is built by showing different facial expressions from a sentence as a form of total communication where facial expressions provide supporting meaning, such as adjectives or descriptions in the information conveyed.
• This dataset can be used for image-based recognition or machine translation research, whether for video data or images that can be extracted from video datasets.

## 2 Background

Several sign language datasets focusing on continuous signing are publicly available. However, a survey [2] reveals that most of these datasets are developed for American Sign Language (ASL) [3,4] and German Sign Language (DGS) [5,6], leaving other sign languages underrepresented. Recent advancements, such as the Turkish Sign Language dataset (AUTSL) [7], highlight the importance of diverse modalities and user-independent benchmarks in addressing real-world challenges. Despite this progress, the Indonesian Sign Language System (*Sistem Isyarat Bahasa Indonesia* or SIBI) lacks comprehensive open datasets. Researchers working on SIBI recognition often had to create their own datasets [[8], [9], [10], [11], [12], [13], [14]], leading to inconsistencies in data collection methods, variations in signer styles, and challenges in ensuring reproducible model performance.

Sign language communication generally relies on hand gestures (manual gestures), but non-manual components such as facial expressions provide additional context [15]. The SIBI framework [1] incorporates Total Communication by integrating both manual and non-manual gestures. SIBI follows Indonesian language grammar unlike other sign languages, which have distinct structures from spoken languages [16]. This adherence introduces complexity, as words in SIBI are formed by combining root word signs and affixes, reflecting the linguistic structure of the Indonesian language.

## 3 Data Description

The SIBI dataset comprises basic signs (including the alphabet, numbers, and affixes) as well as sentences with accompanying facial expressions. This dataset represents an enhancement over the data used in previous research on temporal action segmentation in SIBI [17]. The objectives of the dataset are to:

• Create a dataset of basic SIBI sign gestures, including alphabet, numbers, and affixes in video format, encompassing both static and dynamic signs that are publicly available.
• Provide a dataset of SIBI sentences with seven basic facial expressions, which can be further processed according to the needs of the research to be conducted.
• Encourage researchers to work on the topic of SIBI sign language translation.

Recording was conducted at SLB Negeri 2 Denpasar, Bali, Indonesia. SLB Negeri 2 Denpasar was selected as the data collection site because It is a specialized institution dedicated to the education of students with special needs, particularly those with hearing impairments. In addition to offering a dedicated program for hearing-impaired students, this school also serves as a pilot institution under the Ministry of Education's *Program Sekolah Penggerak*^2^. With its adequate facilities and teachers trained in the Indonesian Sign Language System (SIBI), SLB Negeri 2 Denpasar represents an ideal setting for obtaining high-quality data.

The population in this study consisted of hearing-impaired students without additional special needs (multiple disabilities) who are proficient in SIBI, as well as teachers who instruct hearing-impaired students and possess expertise in SIBI. All hearing-impaired students meeting the specified criteria and providing consent for participation were included in the study. Student selection was conducted based on diagnostic assessments performed by teachers at the beginning of the academic term, ensuring that the students involved possessed the requisite skills to comprehend and utilize SIBI effectively. Teachers included in the study comprised all educators responsible for classes of hearing-impaired students and who demonstrated a comprehensive understanding of SIBI. This approach ensured an accurate representation of the relevant population for research on SIBI. The recording involved 20 volunteers, hereafter referred to as signers, consisting of 10 teachers and 10 hearing-impaired students. Table 1 shows the gender and age of each signer.

**Table 1:** Signer profile.

No of Signer	Gender	Age	No of Signer	Gender	Age
1	Female	39	11	Female	18
2	Female	40	12	Female	17
3	Female	37	13	Female	17
4	Female	29	14	Female	18
5	Female	28	15	Female	20
6	Female	38	16	Male	21
7	Male	29	17	Male	18
8	Female	45	18	Male	18
9	Female	47	19	Female	17
10	Male	35	20	Female	18

**Fig. 1:**
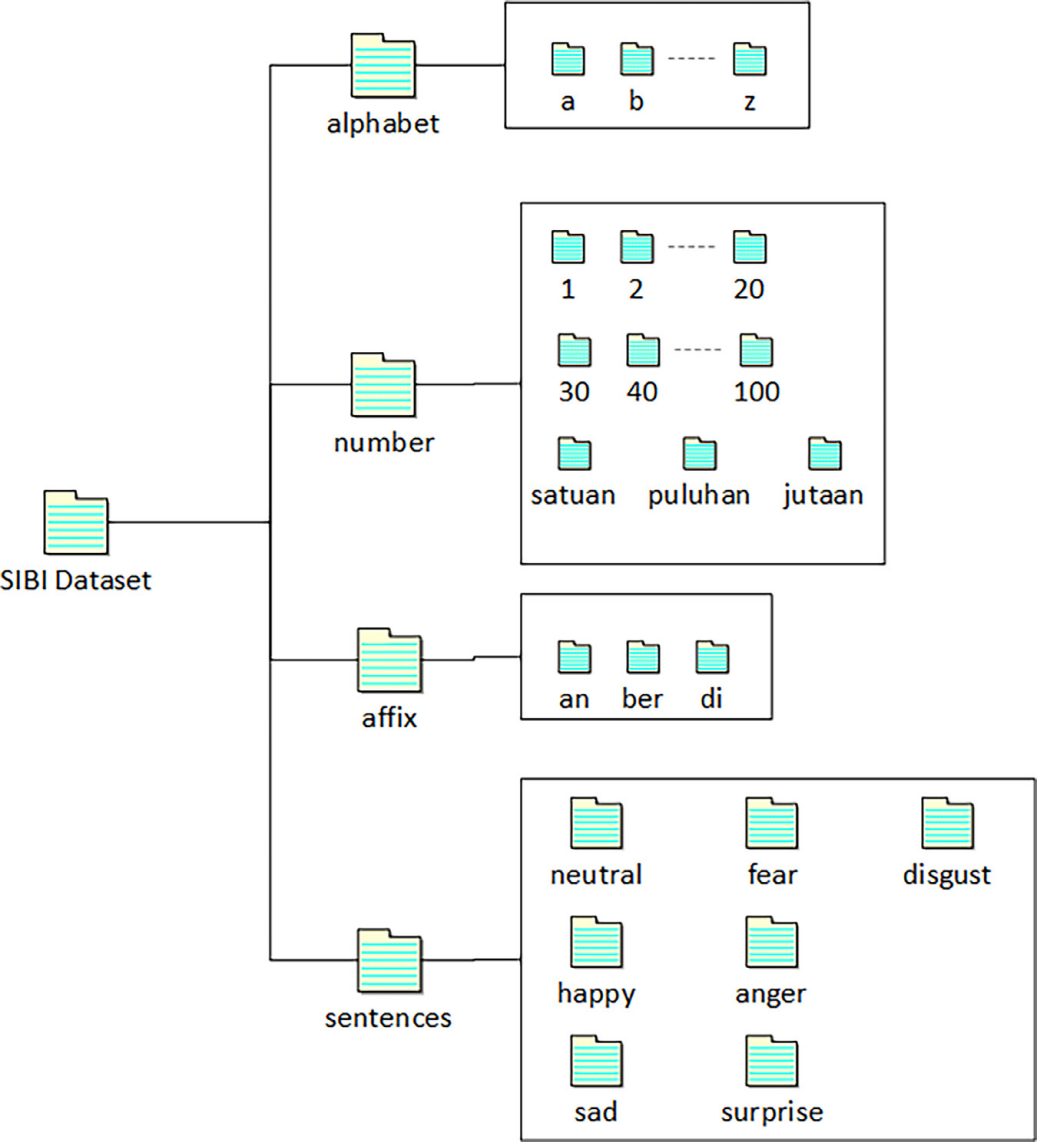
Directory structure.

The dataset consists of 4 main folders: alphabet, number, affix, and sentences with the directory structure shown in Fig. 1. Table 2 shows more details regarding the directory structure and sub-directories shown in Table 2. Each of the 20 signers recorded each alphabet, number, and affix sign. In number signs, the dataset can be divided into static signs, including numbers 1 to 9, and dynamic signs, a combination of several number sign gestures. For example, the number 11 to 19 sign is a unit gesture performed twice. The *seratus* (hundred) sign combines the signs of *satu* (one) and *ratus* (hundred). In addition to the combination of two number signs, some signs are a combination of number signs with affixes, such as the *satuan* (unit) sign, a combination of the number *satu* (one) sign and the affix *-an* sign. Each video file is saved in a name format according to the information contained, such as, signer number, type of sign, and content of the signal. Table 3 specifies the video file naming format in the SIBI dataset.

**Table 2:** Detailed directory structure.

Folder name	Sub-folder Name	No of Videos
Alphabet	a, b, c, d, e, f, g, h, i, j, k, l, m, n, o, p, q, r, s, t, u, v, w, x, y, z	520
Number	1, 2, 3, 4, 5, 6, 7, 8, 9, 10, 11, 12, 13, 14, 15, 16, 17, 18, 19, 20, 30, 40, 50, 60, 70, 80, 90, *seratus, seribu, sejuta, ratus, puluh, ribu, juta, milyar, triliun, satuan, puluhan, ratusan, ribuan, jutaan*	820
Affix	*an, ber, di, i, kah, kan, ke, lah, man, me, nya, pe, pun, se, ter, ti, wan, wati*	360
Sentences	anger, disgust, fear, happy, neutral, sad, surprise	1400

**Table 3:** Video filename format.

Folder Name	Filename format
Alphabet	signer-[no_signer]-alphabet-[content] example: signer-1-alphabet-a
Number	signer-[no_signer]-number-[content] example: signer-1-number-1
Affix	signer-[no_signer]-affix-[content] example: signer-1-affix-an
Sentences	signer-[no_signer]-sentence-[no_sentence]-[emotion] example: signer-1-sentence-1-neutral

The affix directory consists of 18 types of affixes, including prefixes, suffixes, and particles in Indonesian. The sentences directory contains seven sub-directories representing the basic facial expressions of emotions: anger, disgust, fear, happiness, neutral, sadness, and surprise. Each emotion folder contains video recordings of 10 sentences recorded by 20 signers. The 10 sentences were selected from 40 sentences determined in an earlier stage.

Table 4 shows the Indonesian sentences that will be signed into SIBI and their mapping to seven facial expressions. From Table 4, it can be seen that there are sentences that are mapped to several facial expressions/emotions. The mapping is intended to provide examples of differences in facial expressions. Sign gestures conveyed with facial expressions can affect the meaning of the message conveyed. The codes 1-10 in the facial expression columns refer to the sentence number (no_sentence), which is a component of the video file name, as described in Table 3.

**Table 4:** Mapping sentences to facial expressions.

No	Sentences (in Bahasa)	English Translation	Sentence Number						
			Happy	Sad	Anger	Disgust	Fear	Surprise	Neutral
1	*Saya mendapatkan jadwal olah raga sekarang*	I got the sports schedule now	1	1					1
2	*Saya diajak teman jalan-jalan ke pantai jam 1 siang*	My friend invited me to go on a trip to the beach at 1 pm	2	2					
3	*Saya membeli buah di pasar*	I bought fruit at the market	3	3		1		1	2
4	*Saya mendapatkan juara 2*	I got 2nd place	4	4	1				3
5	*Saya mengerjakan tugas kelompok sendiri*	I do group assignments myself	5		2				
6	*Saya berenang di pantai*	I swim at the beach	6				1		
7	*Saya melihat ibu memasak ikan*	I saw mother cooking fish	7			2			4
8	*Saya melihat pak guru memasak kue*	I saw the teacher cooking cakes	8						5
9	*Saya di ajak kakak bersepeda keliling desa*	My older brother invited me to cycle around the village	9					2	
10	*Saya melihat anjing di jalan*	I saw a dog on the street	10				2		
11	*Saya kalah lomba*	I lost the race		5				3	
12	*Anjing saya mati*	My dog died		6				4	
13	*Uang saya hilang*	My money is gone		7				5	
14	*Nilai ku jelek*	My grades are bad		8	3				
15	*Dia memukul saya*	He hit me		9	4		3		
16	*Dia menyobek buku saya*	He tore my book		10	5				
17	*Dia ambil pensilku*	He took my pencil			6				
18	*Dia menjatuhkan makananku*	He dropped my food			7			6	
19	*Dia mendorong saya*	He pushed me			8			7	
20	*Dia mengejek saya*	He mocked me			9				
21	*Dia mencubit saya*	He pinched me			10			8	
22	*Saya menangkap belut*	I caught an eel				3	4		
23	*Sampahnya bau*	The trash smells				4			
24	*Sepatu dia kotor*	His shoes are dirty				5			
25	*Kamar mandi itu kotor*	The bathroom was dirty				6			
26	*Tempat sampah banyak semut*	The trash can have a lot of ants				7			
27	*Saya melihat dia meludah*	I saw him spit				8			
28	*Saya mencium bau badannya*	I smelled his body odor				9		9	
29	*Saya melihat kukunya hitam*	I saw his nails were black				10		10	
30	*Saya melihat petir*	I saw lightning					5		
31	*Saya melihat ada ular masuk kelas*	I saw a snake enter the classroom					6		
32	*Saya tidak mengerjakan tugas*	I didn't do the assignment					7		
33	*Kemarin saya menonton film horor*	Yesterday I watched a horror movie					8		
34	*Saya terlambat sampai di sekolah*	I arrived late at school					9		
35	*Saya melihat tangan dia berdarah*	I saw his hands were bleeding					10		
36	*Saya pulang jam 2 siang*	I come home at 2 pm							6
37	*Saya punya 3 saudara*	I have 3 brothers							7
38	*Saya lahir tahun 2012*	I was born in 2012							8
39	*Apakah kamu punya pulpen?*	Do you have a pen?							9
40	*Selamat pagi, nama saya JINGGA*	Good morning, my name is JINGGA							10

In Bahasa Indonesia, the pronoun *"dia"* is gender-neutral and does not distinguish between male and female, unlike English, which explicitly differentiates between *"he"* and *"she."* This universal usage of *"dia"* reflects the linguistic structure of Bahasa Indonesia, which does not encode gender in third-person pronouns. Indonesian pronouns such as *"dia"* or *"ia"* are inherently neutral and do not convey information about the subject's gender [18]. Similarly, Lyovin et al. [19] highlight that many Austronesian languages, including Bahasa Indonesia, lack gender distinctions in their pronominal systems, emphasizing their neutral nature in gender representation. In this dataset, the pronouns *"he"* or *"she"* in the English translations are provided merely as illustrative tools for readers. The video data in the SIBI dataset adheres strictly to the morphemes of the original sentences in Bahasa Indonesia, where gender is not explicitly marked. The morphemic structure of the sentences is detailed in Table 5, providing a linguistic foundation for understanding how SIBI adheres to the grammar and morphological rules of Bahasa Indonesia.

**Table 5:** Morphological analysis of sentences.

No	Sentences (in Bahasa)	Sign Language Morphemes									
		1	2	3	4	5	6	7	8	9	10
1	*Saya mendapatkan jadwal olah raga sekarang*	*saya*	*me*	*dapat*	*kan*	*jadwal*	*olahraga*	*sekarang*			
2	*Saya diajak teman jalan-jalan ke pantai jam 1 siang*	*saya*	*di*	*ajak*	*teman*	*jalan-jalan*	*ke*	*pantai*	*jam*	*1*	*siang*
3	*Saya membeli buah di pasar*	*saya*	*me*	*beli*	*buah*	*di*	*pasar*				
4	*Saya mendapatkan juara 2*	*saya*	*me*	*dapat*	*kan*	*juara*	*2*				
5	*Saya mengerjakan tugas kelompok sendiri*	*saya*	*me*	*kerja*	*kan*	*tugas*	*kelompok*	*sendiri*			
6	*Saya berenang di pantai*	*saya*	*ber*	*renang*	*di*	*pantai*					
7	*Saya melihat ibu memasak ikan*	*saya*	*me*	*lihat*	*ibu*	*me*	*masak*	*ikan*			
8	*Saya melihat pak guru memasak kue*	*saya*	*me*	*lihat*	*bapak*	*guru*	*me*	*masak*	*kue*		
9	*Saya diajak kakak bersepeda keliling desa*	*saya*	*di*	*ajak*	*kakak*	*ber*	*sepeda*	*keliling*	*desa*		
10	*Saya melihat anjing di jalan*	*saya*	*me*	*lihat*	*anjing*	*di*	*jalan*				
11	*Saya kalah lomba*	*saya*	*kalah*	*lomba*							
12	*Anjing saya mati*	*anjing*	*saya*	*mati*							
13	*Uang saya hilang*	*uang*	*saya*	*hilang*							
14	*Nilaiku jelek*	*nilai*	*aku*	*jelek*							
15	*Dia memukul saya*	*dia*	*me*	*pukul*	*saya*						
16	*Dia menyobek buku saya*	*dia*	*me*	*sobek*	*buku*	*saya*					
17	*Dia ambil pensilku*	*dia*	*ambil*	*pensil*	*aku*						
18	*Dia menjatuhkan makananku*	*dia*	*me*	*jatuh*	*kan*	*makan*	*an*	*aku*			
19	*Dia mendorong saya*	*dia*	*me*	*dorong*	*saya*						
20	*Dia mengejek saya*	*dia*	*me*	*ejek*	*saya*						
21	*Dia mencubit saya*	*dia*	*me*	*cubit*	*saya*						
22	*Saya menangkap belut*	*saya*	*me*	*tangkap*	*belut*						
23	*Sampahnya bau*	*sampah*	*nya*	*bau*							
24	*Sepatu dia kotor*	*sepatu*	*dia*	*kotor*							
25	*Kamar mandi itu kotor*	*kamar*	*mandi*	*itu*	*kotor*						
26	*Tempat sampah banyak semut*	*tempat*	*sampah*	*banyak*	*semut*						
27	*Saya melihat dia meludah*	*saya*	*me*	*lihat*	*dia*	*me*	*ludah*				
28	*Saya mencium bau badannya*	*saya*	*me*	*cium*	*bau*	*badan*	*nya*				
29	*Saya melihat kukunya hitam*	*saya*	*me*	*lihat*	*kuku*	*nya*	*hitam*				
30	*Saya melihat petir*	*saya*	*me*	*lihat*	*petir*						
31	*Saya melihat ada ular masuk kelas*	*saya*	*me*	*lihat*	*ada*	*ular*	*masuk*	*kelas*			
32	*Saya tidak mengerjakan tugas*	*saya*	*tidak*	*me*	*kerja*	*kan*	*tugas*				
33	*Kemarin saya menonton film horor*	*kemarin*	*saya*	*me*	*tonton*	*film*	*horor*				
34	*Saya terlambat sampai di sekolah*	*saya*	*ter*	*lambat*	*sampai*	*di*	*sekolah*				
35	*Saya melihat tangan dia berdarah*	*saya*	*me*	*lihat*	*tangan*	*dia*	*ber*	*darah*			
36	*Saya pulang jam 2 siang*	*saya*	*pulang*	*jam*	*2*	*siang*					
37	*Saya punya 3 saudara*	*saya*	*punya*	*3*	*saudara*						
38	*Saya lahir tahun 2012*	*saya*	*lahir*	*tahun*	*2*	*ribu*	*12*				
39	*Apakah kamu punya pulpen?*	*apa*	*kah*	*kamu*	*punya*	*pulpen*					
40	*Selamat pagi, nama saya JINGGA*	*selamat*	*pagi*	*nama*	*saya*	*J*	*I*	*N*	*G*	*G*	*A*

Table 5 shows a morphological analysis of 40 sentences. At this stage, the morphemes of the sentence composer are deduced, consisting of the root words and affixes. The word *mendapatkan* in sentence 1 is pronounced *me+dapat+kan*, so the word *mendapatkan* is indicated by three signs gestures. The composition of the morphemes forms the sign of the inflectional word based on the rules of its spoken language which distinguishes SIBI from other sign languages and adds complexity in performing its indications. For the sentence “Saya lahir tahun 2012” (I was born in 2012) in line 38 of Table 5, the morphemic breakdown for the year "2012" is performed based on the structure of sign language morphemes. Instead of splitting the number into individual digits such as "2," "0," "1," and "2," the year is segmented into the morphemes *ribu* (thousand) and 12 (twelve), forming a meaningful phrase in the context of SIBI. This approach ensures that the representation aligns with the natural linguistic structure of the Indonesian language, where numbers are often expressed as grouped units rather than isolated digits.

**Fig. 2:**
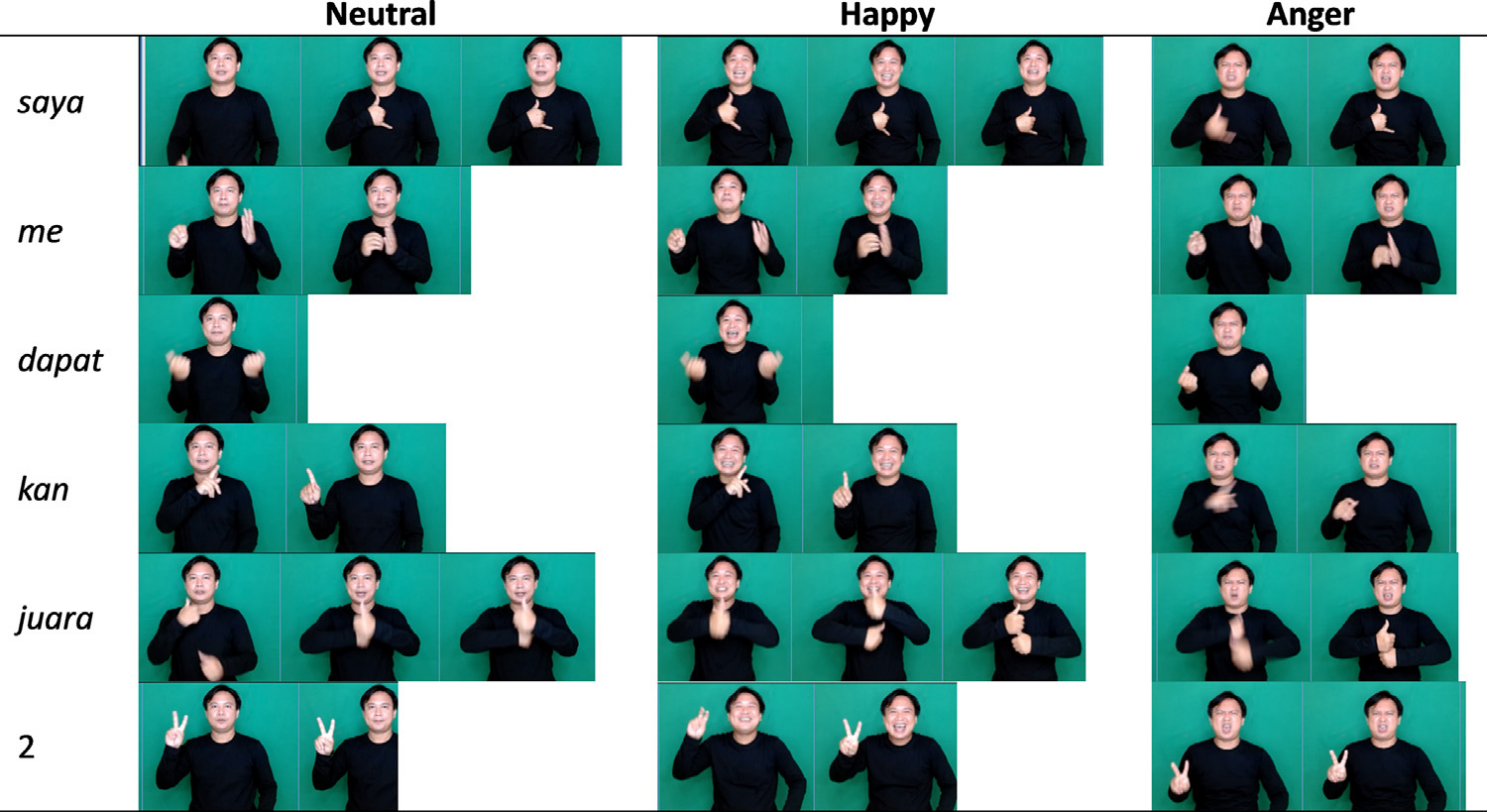
The frame sequence of sentence 4 (*saya me+dapat+kan juara* 2) with different expressions conveyed by the first author as a signer.

Fig. 2 shows the sequence of frames and their grouping based on the word gestures being conveyed. In the picture, the fourth sentence *saya mendapatkan juara 2* (I got 2nd place), presented with a different facial expression. Fig. 2 is presented by the first author with consent to show his face. The different expressions of happiness and anger in the sentence give a different meaning to the message delivered. The SIBI dataset consists of several identical sentences delivered with different facial expressions, as shown in Table 4.

The SIBI dataset, comprising hand gestures, poses, lip movements, and facial expressions from 10 hearing-impaired students and 10 teachers, serves as a comprehensive resource for advancing research in various domains related to sign language and human-computer interaction. One significant application is in gesture recognition, where the dataset can contribute to the development of automated systems for translating SIBI gestures into text or speech, enhancing accessibility for deaf communities. Recent review papers on sign language recognition have emphasized the effectiveness of deep learning models in enhancing gesture recognition accuracy [2,[20], [21], [22], [23], [24], [25]].

The inclusion of diverse facial expressions supports research in affective computing, enabling systems to interpret emotional context alongside gestures, which is critical for improving the accuracy and naturalness of human-computer interactions. Integrating facial expression analysis with gesture recognition has been shown to enhance the performance of sign language recognition systems [[26], [27], [28]].

The dataset's detailed capture of lip movements offers potential for lip-reading research, facilitating multi-modal approaches to speech recognition for sign language users. Combining lip-reading with gesture recognition can lead to more robust and accurate sign language interpretation systems [29]. Furthermore, the combination of visual cues, such as poses and expressions, makes the dataset ideal for developing and validating action recognition models, which are critical in human-computer interaction and video-based activity recognition. Recent advancements in multi-algorithm fusion for gesture recognition highlight the potential of such datasets in improving recognition accuracy [30].

## 4 Materials and Methods

**Fig. 3:**
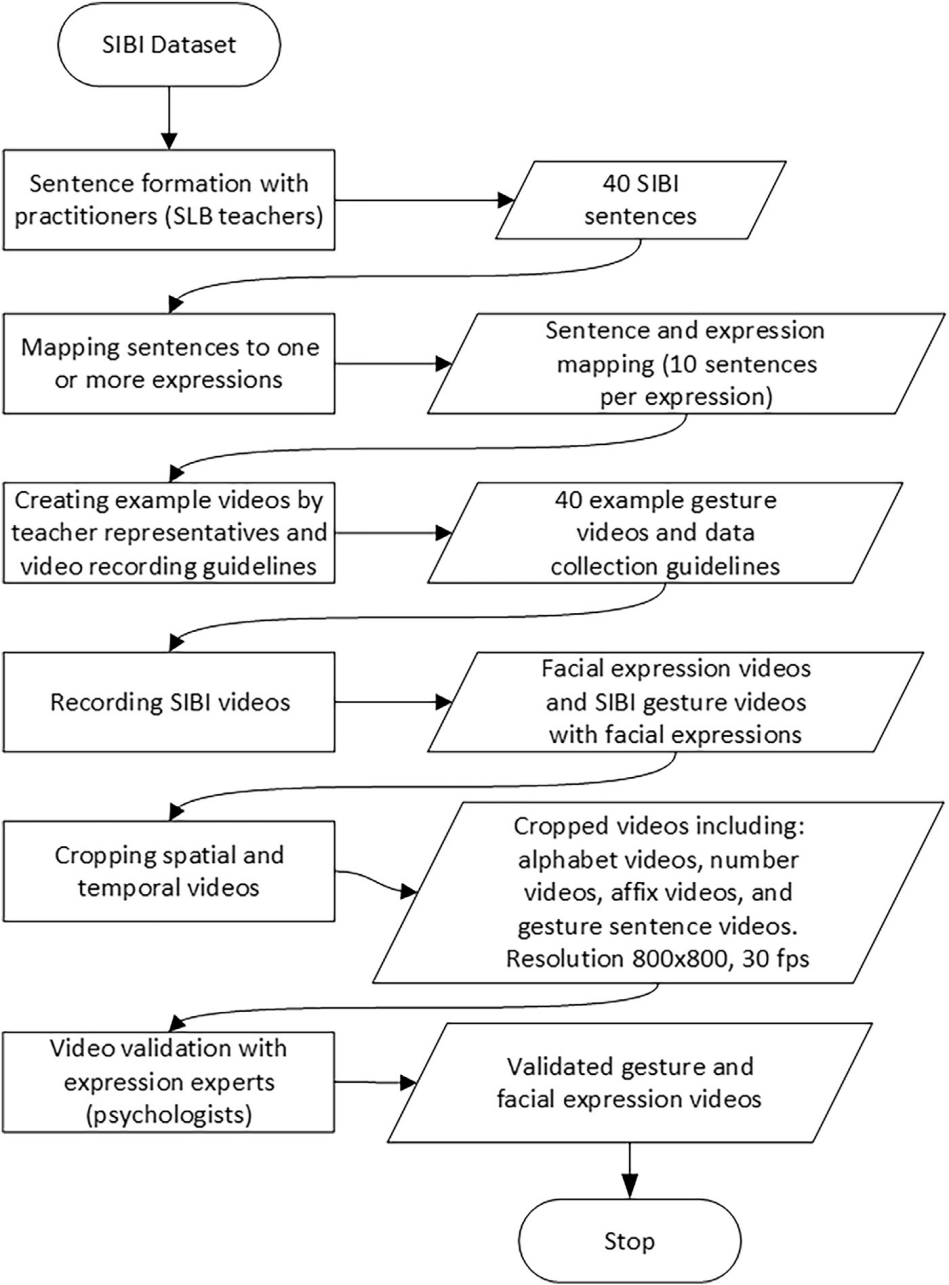
Stages of dataset development.

In general, the SIBI dataset production process goes through four steps: (1) Dataset Preparation, (2) Dataset Gathering, (3) Cropping Images, and (4) Dataset Validation. Each phase is then broken down into several activities and outputs, as shown in Fig. 3.

Several activities are conducted in the Data Preparation phase, including compiling 40 commonly used sentences during learning. Once the 40 sentences are established, the next step is determining the facial expressions that can convey those sign sentences. This phase also creates a recording guide for SIBI videos for 20 signer volunteers, including data collection procedures and example videos of SIBI sign movements for each sentence.

**Fig. 4:**
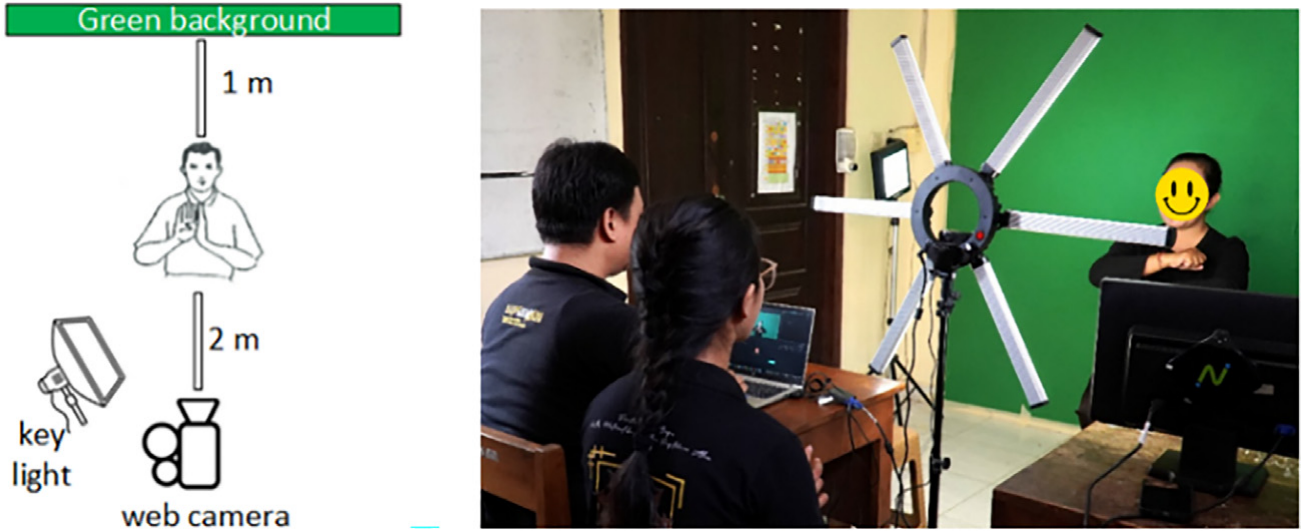
Video recording setup.

In the second phase, a video recording of SIBI is performed. The recording is conducted for the alphabet, numbers, affixes, and several sign sentences with different expressions. The video recording setup is shown in Fig. 4. The signer sits in front of a green screen at a distance of 1 meter. Two meters are separating the signer and the camera. The following are the specifications of the recording equipment:

1. Camera: Logitech C920, resolution 1920 × 1080, frame rate 30 fps.
2. Light: starlight, color temperature 5600K.
3. Background: green.
4. Signer's clothes: long-sleeved black shirt.

Post-recording tasks are completed in phase three. During this stage of the process, videos are cropped to an 800 × 800 resolution at a frame rate of 30 frames per second. The 800 × 800 resolution is chosen to fully display the signer within a single frame, focusing on the upper body above the waist. Additionally, audio is removed from the videos during this stage.

The fourth phase is dataset validation. SIBI sign language experts conduct the initial validation to ensure the signs are accurate according to the SIBI online dictionary. Once the correctness of the sign movements in each recording is confirmed, facial expression validation for the sentence recordings is performed by facial expression experts/psychologists.

## Limitations

This study acknowledges several limitations in the dataset and data collection process:

1. Due to health issues, some signers still wore glasses during the recording sessions, which may have caused light reflections from the lamps and potentially affected the visibility of facial expressions.
2. The videos do not include audio but capture lip movements to support potential lip-reading research.
3. The dataset comprises a limited number of subjects, with an unequal gender distribution between male and female signers.
4. The dataset includes 40 sentences comprising 114 morphemes. These sentences represent various phrases using basic root words, affixes, numbers, and the alphabet. Expanding sentence variations is planned for future work.
5. Recordings were conducted with a green screen background for consistency but may not fully reflect real-world conditions, potentially impacting model generalizability.

Despite these limitations, the dataset provides a foundational resource for SIBI-related research and can be expanded or adapted in future work to address these constraints.

## Ethics Statement

This data collection involved human subjects and was conducted in accordance with the Ethical Clearance Approval on Social Studies and Humanities Research No: 731/KE.01/SK/08/2024, issued by the Ethical Committee on Social Studies and Humanities, National Research and Innovation Agency (BRIN), Indonesia. All data collected from voluntary subjects were presented anonymously. The video recording involving teachers and high school students aged at least 17 years has been approved by the principal of SLB Negeri 2 Denpasar, as evidenced by the signed *Berita Acara Penelitian* (Research Report). Informed consent forms, consent forms from each subject, and parental permission letters (for subjects aged 17 years) are attached to the consent form. In order to pay attention to the protection of personal data showing the face of each subject, we only attach a video of one subject, who is the author, with a disguised face and dataset metadata. Researchers interested in using the dataset can agree and send a Data Use Agreement (DUA) to the data owner.

## CRediT Author Statement

**I Dewa Made Bayu Atmaja Darmawan:** Conceptualization, Methodology, Data Curation, Investigation, Writing - Original Draft. **Linawati:** Supervision, Writing – Review & Editing, Visualization, Resources. **Gede Sukadarmika:** Writing – review & editing, Investigation. **Ni Made Ary Esta Dewi Wirastuti:** Writing – review & editing, Investigation. **Reza Pulungan:** Writing – review & editing, Investigation.

## Data Availability

Mendeley DataIndonesian Sign Language System (SIBI) Dataset (Original data)

## Footnotes

1 https://pmpk.kemdikbud.go.id/sibi/.

## Footnotes

2 *Program Sekolah Penggerak* is an initiative by the Indonesian Ministry of Education aimed at improving the quality of education through transformative practices and capacity building for schools to become role models in student-centered learning.
