# Supplementary material for: Indonesian sign language system (SIBI) dataset: Sentences enhanced by diverse facial expressions for total communication
Source: Data Brief. 2025 May 10;60:111642. doi: 10.1016/j.dib.2025.111642 (PMC12152575; doi:10.1016/j.dib.2025.111642)
Supplement: Supplementary file 1 [file mmc1.pdf]

KEMENTERIAN PENDIDIKAN, KEBUDAYAAN
RISET, DAN TEKNOLOGI
UNIVERSITAS UDAYANA
LEMBAGA PENELITIAN DAN PENGABDIAN KEPADA MASYARAKAT
Jalan Kampus Bukit Jimbaran Badung, Bali
Telepon (0361) 703367 , 704622 , 701954 ext.106
Laman : www.lppm.unud.ac.id ; Email : info-lppm@unud.ac.id

DATA USE AGREEMENT

This is an agreement ("Agreement") between you, the downloader ("Downloader"), and the owner of the materials ("Data Owner") governing the use of the materials ("Materials") to be downloaded.

I. Acceptance of this Agreement

By downloading or otherwise accessing the Materials, the Downloader represents his/her acceptance of the terms of this Agreement.

II. Modification of this Agreement

The Data Owner may modify the terms of this Agreement at any time. However, any modifications to this Agreement will only be effective for downloads subsequent to such modification. No modifications will supersede any previous terms that were in effect at the time of the Downloader's download.

III. Use of the Materials

The use of the Materials is permitted only for non-commercial scientific research. The Data Users must acknowledge the source and background of the data set by citing the data article and any related articles as follows:

1. Darmawan IDMBA, Linawati, Sukadarmika G, Wirastuti NMAED, Pulungan R, Mulyanto, Hariyanti NKD. "Advancing Total Communication in SIBI: A Proposed Conceptual Framework for Sign Language Translation." 2023 International Conference on Smart-Green Technology in Electrical and Information Systems (ICSGTEIS), 2023, p. 23–8. https://doi.org/10.1109/ICSGTEIS60500.2023.10424020.

2. Darmawan IDMBA, Linawati, Sukadarmika G, Wirastuti NMAED, Pulungan R. "Temporal Action Segmentation in Sign Language System for Bahasa Indonesia (SIBI) Videos Using Optical Flow-Based Approach." Jurnal Ilmu Komputer Dan Informasi 2024;17:195–202. https://doi.org/10.21609/jiki.v17i2.1284.

3. Darmawan IDMBA, Linawati, Sukadarmika G, Wirastuti NMAED, Pulungan R; Wirastuti, Ni Made Ary Esta Dewi; Pulungan, Reza (2024), " Indonesian Sign Language System (SIBI) Dataset", Mendeley Data, V3, doi: https://doi.org/10.21609/10.17632/44pbrbsnkh.3.

IV. Restrictions on the Use of the Materials

The Downloader agrees to the following restrictions:

- The Materials will not be used for commercial purposes.
- The Materials will not be shared with third parties without written permission from the Data Owner.
- The Materials will not be used to attempt to re-identify individuals.
- Redistribution of the SIBI dataset (including via Github, Kaggle, Colaboratory, GitCafe, CSDN, etc.) is prohibited.
- Posting SIBI videos/images on the web and social media is prohibited.
- Public exhibition of SIBI videos/images in museums/galleries is prohibited.
- Broadcasting in the mass media (TV shows, films, etc.) is prohibited.

V. Representations and Warranties

THE DATA OWNER REPRESENTS THAT THE DATA OWNER HAS ALL RIGHTS REQUIRED TO MAKE AVAILABLE AND DISTRIBUTE THE MATERIALS. EXCEPT FOR SUCH REPRESENTATION, THE MATERIALS ARE PROVIDED "AS IS" AND "AS AVAILABLE" AND WITHOUT WARRANTY OF ANY KIND, EXPRESS OR IMPLIED, INCLUDING, BUT NOT LIMITED TO, NON-INFRINGEMENT, MERCHANTABILITY, AND FITNESS FOR A PARTICULAR PURPOSE, AND ANY WARRANTIES IMPLIED BY ANY COURSE OF PERFORMANCE OR USAGE OF TRADE, ALL OF WHICH ARE EXPRESSLY DISCLAIMED.

WITHOUT LIMITING THE FOREGOING, THE DATA OWNER DOES NOT WARRANT THAT: (A) THE MATERIALS ARE ACCURATE, COMPLETE, RELIABLE, OR CORRECT; (B) THE MATERIALS FILES WILL BE SECURE; (C)

THE MATERIALS WILL BE AVAILABLE AT ANY PARTICULAR TIME OR LOCATION; (D) ANY DEFECTS OR ERRORS WILL BE CORRECTED; (E) THE MATERIALS AND ACCOMPANYING FILES ARE FREE OF VIRUSES OR OTHER HARMFUL COMPONENTS; OR (F) THE RESULTS OF USING THE MATERIALS WILL MEET THE DOWNLOADER'S REQUIREMENTS. THE DOWNLOADER'S USE OF THE MATERIALS IS SOLELY AT THE DOWNLOADER'S OWN RISK.

VI. Limitation of Liability

IN NO EVENT SHALL THE DATA OWNER BE LIABLE UNDER CONTRACT, TORT, STRICT LIABILITY, NEGLIGENCE, OR ANY OTHER LEGAL THEORY WITH RESPECT TO THE MATERIALS (I) FOR ANY DIRECT DAMAGES, OR (II) FOR ANY LOST PROFITS OR SPECIAL, INDIRECT, INCIDENTAL, PUNITIVE, OR CONSEQUENTIAL DAMAGES OF ANY KIND WHATSOEVER.

VII. Indemnification

The Downloader will indemnify and hold the Data Owner harmless from and against any and all loss, cost, expense, liability, or damage, including, without limitation, all reasonable attorneys' fees and court costs, arising from (i) the Downloader's misuse of the Materials; (ii) the Downloader's violation of the terms of this Agreement; or (iii) infringement by the Downloader or any third party of any intellectual property or other right of any person or entity contained in the Materials. Such losses, costs, expenses, damages, or liabilities shall include, without limitation, all actual, general, special, and consequential damages.

VIII. Dispute Resolution

The Downloader and the Data Owner agree that any cause of action arising out of or related to the download or use of the Materials must commence within one (1) year after the cause of action arose; otherwise, such cause of action is permanently barred.

This Agreement shall be governed by and interpreted in accordance with the laws of the Republic of Indonesia (excluding the conflict of laws rules thereof). All disputes under this Agreement will be resolved in the applicable courts of

Indonesia. The Downloader consents to the jurisdiction of such courts and waives any jurisdictional or venue defenses otherwise available.

IX. Integration and Severability

This Agreement represents the entire agreement between the Downloader and the Data Owner with respect to the downloading and use of the Materials and supersedes all prior or contemporaneous communications and proposals (whether oral, written, or electronic) between the Downloader and the Data Owner with respect to downloading or using the Materials. If any provision of this Agreement is found to be unenforceable or invalid, that provision will be limited or eliminated to the minimum extent necessary so that the Agreement will otherwise remain in full force and effect and enforceable.

X. Miscellaneous

The Data Owner may assign, transfer, or delegate any of its rights and obligations hereunder without consent. No agency, partnership, joint venture, or employment relationship is created as a result of the Agreement, and neither party has any authority of any kind to bind the other in any respect outside of the terms described within this Agreement. In any action or proceeding to enforce rights under the Agreement, the prevailing party will be entitled to recover costs and attorneys' fees.

Data Owner:

Darmawan, I Dewa Made Bayu Atmaja
Email: dewabayu@unud.ac.id

KEMENTERIAN PENDIDIKAN, KEBUDAYAAN
RISET, DAN TEKNOLOGI
UNIVERSITAS UDAYANA
LEMBAGA PENELITIAN DAN PENGABDIAN KEPADA MASYARAKAT
Jalan Kampus Bukit Jimbaran Badung, Bali
Telepon (0361) 703367 , 704622 , 701954 ext.106
Laman : www.lppm.unud.ac.id ; Email : info-lppm@unud.ac.id

REQUEST FORM FOR SIBI DATASET

**Basic Information**	
Name of requesting person	
Academic status<br>(e.g. Prof, PhD, PhD student, etc.)	
Gender	Male / Female
Email Address	
Name of Institution	
Country	
**Objective of SIBI Dataset Usage**	
Purpose of Usage	
Research topic	
Are you agree with the Data Usage Agreement (DUA)?	**Yes** / ~~No~~
Date	Signature<br><br>________________________
Note: send it back to the data owner (dewabayu@unud.ac.id); students requesting the dataset must accompany this form with a letter of support/recommendation from their supervisor and	
